# Laboratory Confirmation of Buruli Ulcer Disease in Togo, 2007–2010

**DOI:** 10.1371/journal.pntd.0001228

**Published:** 2011-07-19

**Authors:** Gisela Bretzel, Kristina Lydia Huber, Basil Kobara, Marcus Beissner, Ebekalisai Piten, Karl-Heinz Herbinger, Franz Xaver Wiedemann, Komi Amekuse, Abiba Banla Kere, Kerstin Helfrich, Erna Fleischmann, Thomas Löscher, Adolf Diefenhardt, Jörg Nitschke

**Affiliations:** 1 Department of Infectious Diseases and Tropical Medicine (DITM), University Hospital, Ludwig-Maximilians University (LMU), Munich, Germany; 2 German Leprosy and Tuberculosis Relief Association (DAHW), Würzburg, Germany; 3 Centre Hospitalier Régionale Tsévié (CHR), Tsévié, Togo; 4 German Leprosy and Tuberculosis Relief Association (DAHW), Togo Office, Lomé, Togo; 5 Institut National d'Hygiène (INH), Lomé, Togo; 6 Programme National de Lutte contre L'Ulcère de Buruli – Lèpre et Pian (PNLUB-LP), Lomé, Togo; University of Tennessee, United States of America

## Abstract

**Background:**

Since the early 1990s more than 1,800 patients with lesions suspicious for Buruli ulcer disease (BUD) have been reported from Togo. However, less than five percent of these were laboratory confirmed. Since 2007, the Togolese National Buruli Ulcer Control Program has been supported by the German Leprosy and Tuberculosis Relief Association (DAHW). Collaboration with the Department for Infectious Diseases and Tropical Medicine (DITM), University Hospital, Munich, Germany, allowed IS*2404* PCR analysis of diagnostic samples from patients with suspected BUD during a study period of three years.

**Methodology/Principal Findings:**

The DAHW integrated active BUD case finding in the existing network of TB/Leprosy Controllers and organized regular training and outreach activities to identify BUD cases at community level. Clinically suspected cases were referred to health facilities for diagnosis and treatment. Microscopy was carried out locally, external quality assurance (EQA) at DITM. Diagnostic samples from 202 patients with suspected BUD were shipped to DITM, 109 BUD patients (54%) were confirmed by PCR, 43 (29.9%) by microscopy. All patients originated from Maritime Region. EQA for microscopy resulted in 62% concordant results.

**Conclusions/Significance:**

This study presents a retrospective analysis of the first cohort of clinically suspected BUD cases from Togo subjected to systematic laboratory analysis over a period of three years and confirms the prevalence of BUD in Maritime Region. Intensified training in the field of case finding and sample collection increased the PCR case confirmation rate from initially less than 50% to 70%. With a PCR case confirmation rate of 54% for the entire study period the WHO standards (case confirmation rate ≥50%) have been met. EQA for microscopy suggests the need for intensified supervision and training. In January 2011 the National Hygiene Institute, Lomé, has assumed the role of a National Reference Laboratory for PCR confirmation and microscopy.

## Author Summary

Buruli ulcer disease (BUD) is an emerging disease particularly affecting children under the age of 15 years. Due to scarring and contractures BUD may lead to severe functional disability. Introduction of antimycobacterial treatment necessitated the laboratory confirmation of BUD, and WHO recommends confirmation of at least 50% of patients with suspected BUD by polymerase chain reaction (PCR). In Togo, cases have been reported since the early 1990s. However, less than five percent were laboratory confirmed. Since 2007, the German Leprosy and Tuberculosis Relief Organization (DAHW) has supported the Togolese National Buruli Ulcer Control Program in the area of training, treatment and laboratory confirmation of BUD. In close collaboration of DAHW and the Department for Infectious Diseases and Tropical Medicine, University Hospital, Munich (DITM), diagnostic samples from Togolese patients with suspected BUD were subjected to PCR. Out of 202 suspected BUD cases 109 BUD patients (54%) were PCR confirmed over a period of three years. Whereas the PCR case confirmation rate initially was below 50%, intensified training measures for health staff in the field of clinical diagnosis and collection of diagnostic samples ultimately resulted in 69% PCR confirmed cases. Our findings confirm the prevalence of BUD in Maritime Region.

## Introduction

Buruli ulcer disease (BUD), caused by *Mycobacterium ulcerans*, has become the third most common mycobacterial disease after tuberculosis and leprosy. Cases have been reported from more than 30 countries worldwide with a focus on West Africa. The disease mainly affects impoverished inhabitants of remote rural areas, particularly children under the age of 15 years [1].

BUD involves the skin and the subcutaneous adipose tissue. The disease starts as painless papule, plaque or nodule that evolves into large painless ulcerations with characteristically undermined edges and may be accompanied by edema of the surrounding skin. Large ulcers may affect the subjacent bones resulting in osteomyelitis [1]. Self-healing processes may lead to scarring and contractures. Though mortality is low, morbidity and subsequent functional disability are severe [2]–[5].

Since 2004, antimycobacterial treatment (if necessary followed by surgical intervention) has been considered the treatment of choice [1], [6]–[9]. With the introduction of antimycobacterial treatment, the laboratory confirmation of clinically suspected BUD cases became crucial for the clinical management of BUD. Therefore, WHO strongly recommends collection of diagnostic samples from all clinically suspected BUD cases [1], [10], [11].

Currently available diagnostic laboratory tests include microscopic examination, culture, IS*2404* polymerase chain reaction (PCR) and histopathological analysis. Swab samples, fine needle aspirates (FNA), punch biopsies and surgical biopsies have been used as diagnostic specimens. Microscopy is considered a suitable first line diagnostic test to be applied in field settings. PCR provides the highest sensitivity, therefore is regarded the method of choice for laboratory confirmation as well as sufficient evidence to commence antimycobacterial treatment. WHO encourages all endemic countries to ensure that at least 50% of all cases are confirmed by PCR [1], [11]–[14].

Since the early 1990s patients with lesions clinically suspicious for BUD have been treated in Togolese hospitals. The first two laboratory-confirmed and well documented BUD patients from Togo were described in 1996 by Meyers and colleagues [15]. A case series of 21 clinically diagnosed BUD patients hospitalized from 1994 through 1996 was reported by Songné [16]. A hospital based study conducted from 2000 through 2001 identified 180 patients with suspected BUD, 23 out of those were laboratory confirmed [17]. According to data available at the Togolese Ministry of Health, from 1996 through 2004 more than 100 suspected BUD cases were notified, and approximately 20% of these were PCR confirmed at the Institute for Tropical Medicine, Antwerp, Belgium. In 2004, a nationwide survey detected 1505 suspected cases of BUD [“Politique Nationale de Lutte contre L'Ulcère de Buruli.” Ministère de la Santé, République Togolaise, Lomé 2007; 18].

In 1999, Togo established its National Buruli Ulcer Control Program (Programme National de Lutte contre L'Ulcère de Buruli [PNLUB], since 2010: Programme National de Lutte contre L'Ulcère de Buruli – Lèpre et Pian [PNLUB-LP]). Initially limited resources hampered the progress of program activities, however, collaboration with non-governmental organizations (Handicap International [HI], France; German Leprosy and Tuberculosis Relief Association [DAHW], Germany) enhanced the efficiency of BUD control. In 2007, a five year strategic plan was developed to intensify treatment, case detection, laboratory confirmation and surveillance of BUD, initially focusing on Maritime and Central Region. The Centre Hospitalier Régionale (CHR) Tsévié, Maritime Region, was appointed National Reference Centre for BUD in Togo, and recently the Centre Hospitalier Préfectoral (CHP) Sotouboua, Central Region, was turned into an outpost of the National Reference Centre. The DAHW in particular supports training, treatment and laboratory confirmation of patients with suspected BUD [“Plan Stratégique de Lutte contre L'Ulcère de Buruli, 2008–2012”. Ministère de la Santé, République Togolaise, Lomé 2007; 18].

Whereas microscopic analysis has been locally carried out at CHR Tsévié, facilities for the diagnostic IS*2404* PCR were not available before 2011. Therefore, in 2007 DAHW and the Department of Infectious Diseases and Tropical Medicine, University Hospital, Ludwig-Maximilians University, Munich, Germany (DITM) began a collaboration to analyze diagnostic samples from patients with suspected BUD by PCR at DITM. This study presents a retrospective analysis of the laboratory results of the first cohort of suspected BUD cases from Togo subjected to systematic laboratory analysis. The results of this study also give proof that a collaborative effort of local and international partners allows the successful implementation of a diagnostic system within a relatively short period of time.

## Methods

### Ethics Statement

Laboratory confirmation and treatment of BUD patients was covered by a skeleton agreement between the DAHW and the Ministry of Health, Togo. As all activities fall under routine patient management, ethical clearance by the Committee of Bioethics in Research, Ministry of Health, Togo, was not required. In accordance with standard practice customary in Togo, from 2007 through 2008 patients with suspected BUD were verbally informed on the need for collection of diagnostic samples and treatment, and verbal consent was obtained from the patients. In 2009, the PNLUB-LP introduced informed consent forms. Written informed consent (signature or thumb print, in case of minors given by legal representatives) was obtained from the majority of patients with suspected BUD attending CHR Tsévié. Publication of pseudonymized data and results obtained during the study period was authorized by the PNLUB-LP.

### Case Finding

To integrate active BUD case finding into the existing Togolese network of TB and Leprosy District and Regional Controllers (Contrôleur Lèpre–TB–Buruli, CLT), the DAHW conducted two initial training workshops for CLT and health staff at CHR Tsévié and CHP Sotouboua in 2007, followed by regular re-training from 2008 through 2010 (four workshops in Maritime Region, one workshop in Central and Kara Region each). Supported by CHR Tsévié hospital staff, the CLT teams conducted quarterly sensitization campaigns and outreach activities to identify cases at community level under coordination of the PNLUB-LP. Clinically suspected BUD cases were referred to peripheral health posts (Unité de Soins Périphérique, USP), CHP Sotouboua or CHR Tsévié for collection of diagnostic samples and treatment. Passive case finding included patients presenting at BUD treatment centers (USPs, CHR-Tsévié and CHP Sotouboua).

### Study Population

From September 2007 through August 2010, 202 suspected BUD cases from three different study sites in Togo (CHR Tsévié, Maritime Region, n = 187; CHP Sotouboua, Central Region, n = 14, USP Agbetiko, Maritime Region, n = 1) were included in the study (table 1).

**Table 1:** Case confirmation rates.

Type of lesion^a^	Study site	Suspected cases	MIC^b^			PCR^c^		
			Confirmed cases [N]	Suspected cases subjected to MIC [N]	Case confirmation rate (%)	Confirmed cases [N]	Suspected cases subjected to PCR [N]	Case confirmation rate (%)
**Non-ulcerative**	Tsévié	49	9	23	(39.1)	38	49	(77,6)
	Sotouboua	2	NA^d^	NA	NA	0	2	(0.0)
	Agbetiko	0	NA	NA	NA	NA	NA	NA
	Total	51	9	23	(39.1)	38	51	(74.5)
**Ulcerative**	Tsévié	138	34	120	(28.3)	71	138	(51.4)
	Sotouboua	12	0	1	(0.0)	0	12	(0.0)
	Agbetiko	1	NA	NA	NA	0	1	(0.0)
	Total	151	34	121	(28.1)	71	151	(47.0)
**All**		202	43	144	(29.9)	109	202	(54.0)

Table 1 describes the case confirmation rates, i.e. the number of laboratory confirmed BUD cases divided by the total number of patients with suspected BUD (suspected cases) of whom samples were subjected to a certain diagnostic test; diagnostic samples from 202 suspected BUD cases (suspected cases) from three study sites in Togo (CHR Tsévié, CHP Sotouboua, USP Agbetiko) were collected within three years (September 2007 through August 2010);

a Non-ulcerative lesions: FNA (fine needle aspiration) samples, punch biopsy samples and surgical biopsy samples were analyzed; ulcerative lesions: swab samples, FNA (fine needle aspiration) samples, punch biopsy samples and surgical biopsy samples were analyzed;

b Test: MIC, microscopic examination for the detection of acid fast bacilli; swab samples and FNA samples were analyzed;

c Test: PCR, polymerase chain reaction, gel-based IS*2404* PCR; swab samples, FNA samples, punch biopsy samples and surgical biopsy samples were analyzed;

d NA, not available;

### Diagnostic Specimens

Diagnostic samples were collected according to standardized procedures which have been developed in the context of previous studies on laboratory diagnosis of BUD in Ghana [13], [19]–[22]. Briefly, swabs were taken by circling the entire undermined edges of ulcerative lesions. Three millimeter punch biopsies and surgical biopsies with a maximum size of 10×10 mm were taken from the center of non-ulcerative lesions or from undermined edges of ulcerative lesions including necrotic tissue. FNA was performed with 21-gauge needles by trans-dermal aspiration. For non-ulcerative lesions, the needle was inserted into the center of the lesion, for ulcerative lesions, FNA was performed with a maximal distance of 1–2 cm from the margins of the ulcers. If collected from surgical patients, all samples were taken under general anesthesia. Swab samples were collected throughout the entire study period. Most surgical biopsy samples were collected during the first six months of the study period, and then gradually replaced by FNA and punch biopsy samples which were introduced in the first half of 2008.

To facilitate sampling, standardized specimen collection bags including swabs, biopsy punches, syringes and needles, containers with transport media (700 µl CLS® [cell lysis solution, Qiagen, Hilden, Germany] for PCR samples) and data entry forms (BU01 form [1] and a specific laboratory data entry form) were provided to the study sites. Table 2 shows the different types of samples collected according to type of lesion and type of treatment (surgical, non-surgical). However, it was not always possible to collect complete sets of specimens.

**Table 2:** Diagnostic specimens and transport media.

Type of Treatment	Type of lesion	Diagnostic test	Transport medium	Swab	FNA^a^	Punch biopsy	Surgical biopsy
**Surgical**	Non-ulcerative	MIC^b^	NA^c^	NA	yes	NA	NA
		PCR^d^	CLS^e^	NA	yes	yes	yes
	Ulcerative	MIC	NA	yes	yes	NA	NA
		PCR	CLS	yes	yes	yes	yes
**Non-surgical**	Non-ulcerative	MIC	NA	NA	yes	NA	NA
		PCR	CLS	NA	yes	yes	NA
	Ulcerative	MIC	NA	yes	yes	NA	NA
		PCR	CLS	yes	yes	yes	NA

Table 2 describes diagnostic specimens and transport media according to diagnostic tests, type of lesion and type of treatment.

a FNA, fine needle aspiration;

b MIC, microscopic examination for the detection of acid fast bacilli;

c NA, not applicable;

d PCR, IS*2404* gel-based polymerase chain reaction;

e CLS, cell lysis solution (Qiagen, Germany).

### Diagnostic Methods and Laboratories

As shown in table 3, swab (n = 115) and FNA samples (n = 115) were subjected to Ziehl-Neelsen smear microscopy at CHR Tsévié and one swab sample was tested at CHP Sotouboua [23]. For external quality assurance (EQA) of microscopic analysis, 85 stained slides were sent to DITM. PCR samples (swabs, n = 152; FNA, n = 167; punch biopsies, n = 172 surgical biopsies, n = 51) with corresponding laboratory data and BU01 forms were shipped to DITM by courier service on a quarterly basis and subjected to gel based IS*2404* PCR (primers MU5 and MU6) according to standardized procedures [13], [21], [24]–[26]. To assure that no contamination occurred during extraction and PCR, extraction controls and negative run controls were processed with each extraction procedure and each PCR.

**Table 3:** Positivity rates.

Number of positive samples/total number of samples tested [N(%)]									
Type of lesion	Study site	Swab		FNA^a^		Punch biopsy		Surgical biopsy	
		MIC [N(%)]^b^	PCR [N(%)]^c^	MIC [N(%)]	PCR [N(%)]	MIC [N(%)]	PCR [N(%)]	MIC [N(%)]	PCR [N(%)]
**Non-ulcerative**	Tsévié	ND^d^	ND	9/23 (39.1)	27/49 (55.1)	NA^e^	32/50 (64.0)	NA	2/3 (66.7)
	Sotouboua	ND	ND	NA	0/1 (0)	NA	NA	NA	0/2 (0)
	Agbetiko	ND	ND	NA	NA	NA	NA	NA	NA
	Total	ND	ND	9/23 (39.1)	27/50 (54.0)	NA	32/50 (64.0)	NA	2/5 (40.0)
**Ulcerative**	Tsévié	27/115 (23.5)	63/142 (44.4)	31/92 (33.7)	45/111 (40.5)	NA	49/121 (40.5)	NA	12/44 (27.3)
	Sotouboua	0/1 (0.0)	0/9 (0.0)	NA	0/5 (0.0)	NA	0/6 (0.0)	NA	0/2 (0.0)
	Agbetiko	NA	0/1 (0.0)	NA	0/1 (0.0)	NA	0/2 (0.0)	NA	NA
	Total	27/116 (23.3)	63/152 (41.5)	31/92 (33.7)	45/117 (38.5)	NA	49/129 (38.0)	NA	12/46 (26.1)
**All**		27/116 (23.3)	63/152 (41.5)	40/115 (34.8)	72/167 (43.1)	NA	81/179 (45.3)	NA	14/51 (27.5)

Table 3 describes the positivity rates, i.e. the number of positive samples divided by the total number of samples tested, of microscopy and IS*2404* gel-based polymerase chain reaction per type of lesion and type of sample; diagnostic samples from 202 patients with suspected BUD from three study sites in Togo (CHR Tsévié, CHP Sotouboua, USP Agbetiko) were collected within three years (September 2007 through August 2010);

a FNA, fine needle aspiration;

b MIC, microscopic examination for the detection of acid fast bacilli;

c PCR, IS*2404*, gel-based polymerase chain reaction;

d ND, not done;

e NA, not available;

The turnaround time between shipment of samples and availability of results averaged approximately two weeks. Results were communicated by email to DAHW and distributed to the hospitals.

### Statistical Analysis

Clinical and epidemiological information derived from laboratory data entry and BU01 forms as well as diagnostic results obtained at DITM and CHR Tsévié were stored in a database (Access 2003, Microsoft Cooperation, Redmond, WA). For analysis, the study period was divided into three observation periods (September 2007 through August 2008, September 2008 through August 2009, September 2009 through August 2010), for clarification selected data are also indicated per calendar year. Beside epidemiological data, the analysis included case confirmation rates (number of laboratory confirmed BUD patients divided by the total number of suspected BUD cases included in the study) per diagnostic test, and positivity rates (number of positive samples divided by the total number of samples tested) per sample type and diagnostic test. Approximative tests (χ2-tests) and t-tests as parametric tests were conducted using Stata software, version 9.0 (Stata Corporation, College Station, TX) and EpiInfo, version 3.3.2 (Centers for Disease Control and Prevention, CDC, Atlanta, GA).

## Results

### Diagnostic Samples

206 sets of specimens from 202 suspected BUD cases were collected for laboratory confirmation. Fifty-one suspected cases (25.2%) had non-ulcerative lesions, 151 (74.8%) had ulcerative lesions. Four suspected cases (2.0%) had two lesions. From 13 of the 202 study participants 13 sets of follow-up specimens were available.

The patients with suspected BUD originated from ten districts in three regions (Maritime, Central and Plateaux). Most of the suspected cases (82.2%) were detected in districts Zio (n = 89 [44.1%]) and Yoto (n = 77 [38.1%]) of Maritime Region. The age range of the suspected cases was 1–72 years (mean = 24.8 years, median = 17 years) and 39.6% of the suspected cases were in age group 5–14 years, 114 of the suspected cases (56.4%) were male.

### Laboratory Confirmed BUD Cases

Out of the 202 patients with suspected BUD 109 were laboratory confirmed as BUD patients. Out of them 43 (39.5%) were confirmed by two and 66 (60.6%) by at least one positive laboratory test. Out of the 13 study participants followed over time twelve were laboratory confirmed at their first presentation at hospital (also the second sample collection rendered positive results). For one of the 13 study participants followed over time neither the first nor the second sample collection rendered positive results.

The overall case confirmation rate by PCR was 54.0% (109/202), and 29.9% (43/144) by microscopy (table 1). Among the 51 suspected BUD cases with non-ulcerative lesions, 38 (74.5%) were confirmed by a positive tissue PCR result (positive FNA PCR result 26/47 [55.3%], positive punch biopsy PCR result 30/44 [68.2%], positive surgical biopsy PCR result 2/3 [66.7%]). FNA and punch biopsy samples were available from 33 out of these 51 suspected cases, thus a comparison of the PCR case confirmation rates for both types of samples was possible. Among these 33 patients with suspected BUD, the case confirmation rate for punch biopsy samples (30/33; 90.9%) was significantly higher than for FNA samples (23/33; 69.7%) (p = 0.03). For 20/33 (60.6%) of these suspected cases both samples had a positive PCR result, 3/33 (9.1%) were confirmed by FNA PCR only, for 10/33 (30.3%) the diagnosis was established by a positive punch biopsy PCR result only, i.e. the additional diagnostic yield of punch biopsy samples for patients with non-ulcerative lesions was 30.3%.

Among the 151 suspected cases with ulcerative lesions, 71/151 (47.0%) were PCR confirmed (table 1). Out of these, 51/131 (38.9%) were confirmed by a positive swab PCR result, and 56/130 (43.1%) had a positive tissue PCR result (positive FNA PCR result 39/104 [37.5%], positive punch biopsy result 39/95 [41.1%], positive surgical biopsy result 4/18 [22.2%]). All types of samples were available from 41 out of these 151 suspected cases, thus a comparison of the PCR case confirmation rates for swab, FNA and punch biopsy samples was possible. Among these 41 suspected cases there was no significant difference in case confirmation rates 31/41 [75.6%] for swab samples, 36/41 [87.8%] for FNA samples and 36/41 [87.8%] for punch biopsy samples (p = 0.22).

The positivity rates for microscopy and PCR per type of specimen are shown in table 3.

EQA for microscopy resulted in 23/37 (62.2%) concordant results, 14 slides (37.8%) were false negative.

### Epidemiological Baseline Data of Confirmed BUD Cases

**Figure 1:**
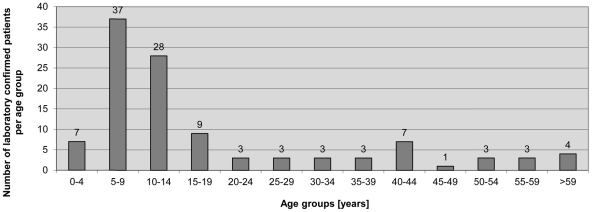
Age distribution of 109 laboratory-confirmed BUD patients. For all patients the age was known and 65 (59.6%) of them were in age group 5–14 years. The age range was 2–60 years with a mean of 17.3 years and the median was 12 years.

Out of the 109 laboratory confirmed BUD patients, 38 (34.9%) had non-ulcerative, 71 (65.1%) had ulcerative lesions, 57 (52.3%) were male, and 65 (59.6%) of them were in age group 5–14 years (age range 2–60 years, mean 17.3 years, median 12 years) (figure 1).

The confirmed BUD patients originated from five districts of Maritime Region (Zio, n = 51; Yoto, n = 49; Vo, n = 5, Golfe, n = 1; Avé, n = 1).

In 90.8% (99/109) the lesions were located on limbs or shoulders. None of the sides was significantly more affected (right side, n = 51 and left side, n = 48).

For all 109 confirmed BUD patients the lesion sizes were known and the lesions were distributed according to WHO categories as follows [1]: category I (single lesion <5 cm in diameter), n = 43 (39.4%); category II (single lesion between 5 and 15 cm in diameter), n = 41 (37.6%); category III (single lesion >15 cm in diameter, multiple lesions, osteomyelitis), n = 25 (22.9%).

Among the BUD patients originating from Maritime Region, five pairs of siblings (two individuals each) were identified, three pairs of siblings developed BUD at the same time. Three pairs of siblings originated from the district of Yoto, two from the district of Zio, all affected families lived close to flowing water bodies (Haho River, Lili River).

### Development of PCR Case Confirmation Rates from 2007 through 2010

**Figure 2:**
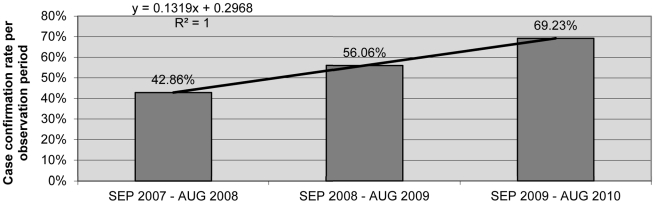
Case confirmation rate per observation period. The PCR case confirmation rate was 36/84 (42.86%) in the first observation period (September 2007–August 2008), 37/66 (56.06%) in the second observation period (September 2008–August 2009) and 36/52 (69.23%) in the third observation period (September 2009–August 2010). The case confirmation rate increased during the three observation periods with a definite trend (coefficient of determination, R^2^ = 1).

The PCR case confirmation rate increased with a definite trend from 42.9% (36/84) to 69.2% (36/52) (coefficient of determination, R^2^ = 1) from the first through the third observation period (figure 2). Calculated per calendar year, the PCR case confirmation rate was 41.7% (10/24) in 2007, 45.8% (38/83) in 2008, 58.9% (33/56) in 2009, and 71.8% (28/39) in 2010 (data not shown).

## Discussion

This study describes the results of a collaborative approach to implement systematic laboratory confirmation of BUD in Togo. Whereas previous data reported from Togo were largely based on clinical observations, this study proves the prevalence of laboratory confirmed BUD cases in Maritime Region. From 2007 through 2010 out of 202 suspected BUD cases 109 BUD patients were PCR confirmed, which equals an overall PCR case confirmation rate of 54%. During the decade after the description of the first two laboratory confirmed BUD patients in 1996 [15], more than 1,800 (in most cases clinically suspected but not laboratory confirmed) BUD cases were reported from Togo [“Politique Nationale de Lutte contre L'Ulcère de Buruli.” Ministère de la Santé, République Togolaise, Lomé 2007; 16–18]. As recently as 2007, the initiation of a close collaboration between PNLUB-LP and several non-governmental organizations as well as the establishment of the National Reference Centre for BUD at CHR Tsévié, laid the foundations for intensified BUD control activities. In accordance with the objectives for BUD control as defined by the Togolese Health Authorities, emphasis was given to early case detection and laboratory confirmation of cases [“Plan Stratégique de Lutte contre L'Ulcère de Buruli, 2008–2012”, Ministère de la Santé, République Togolaise, Lomé 2007; 18]. A collaborative project on laboratory confirmation of patients with suspected BUD conducted by DAHW and DITM allowed for the first time continuous data acquisition over a period of three years.

The strategies applied for collection of diagnostic samples and data management were originally developed in the context of an EC funded research project (project no. INCO-CT-2005-015476-BURULICO) conducted in Ghana [13], [19]–[22]. Visits of DAHW staff at the Kumasi Centre for Collaborative Research in Tropical Medicine (KCCR), Kumasi, Ghana, proved to be instrumental in adopting these procedures for the implementation of laboratory confirmation of BUD in Togo, thus provide an example for efficient South-South collaboration in the area of disease control.

Initially the PCR case confirmation rate was below 50%. However, for the second (56.1%) and third (69.2%) observation period as well as for the entire study period (54.0%) the WHO criteria for PCR case confirmation rates have been met [1], [11]. These findings are mainly attributable to the intensified and regular training activities for CLTs and health staff in the field of clinical diagnosis and collection of diagnostic samples conducted by DAHW.

As far as punch biopsies are concerned, meanwhile broad consensus has been reached that FNA are equal to punch biopsies for most diagnostic applications, and – in the interest of the patients - the use of punch biopsies should be restricted to a minimum [22], [27]–[30]. Whereas the recently published studies on FNA provide details on diagnostic sensitivities of laboratory analysis of various sample types, the data obtained from the Togolese cohort of patients with suspected BUD focus on case confirmation rates. Among the suspected BUD cases with non-ulcerative lesions from Togo the case confirmation rate for punch biopsy samples was significantly higher than for FNA samples. Moreover, PCR analysis of punch biopsy samples allowed the confirmation of 30% additional patients that were not detected by PCR of FNA samples. These findings suggest that at this point in time replacement of punch biopsies by FNA for suspected BUD cases with non-ulcerative lesions is not advisable. Upcoming training activities have to focus on improvement of FNA sample collection techniques and the use of punch biopsies should be maintained until analysis of diagnostic results provides sufficient evidence that no more case are missed if FNA is applied.

A number of limitations of this study need to be mentioned. During the study period most training workshops were held in Maritime Region – which is reflected in a continuous improvement of the quality of samples and data obtained from the catchment area of CHR Tsévié. In contrast, all diagnostic samples sent from Central Region were negative, therefore this study did not succeed to confirm the prevalence of BUD outside of Maritime Region. Further attempts to verify if the disease occurs in other regions of the country require intensified training in the field of clinical diagnosis and collection of diagnostic samples in the respective areas.

Furthermore, this study did not use specific questionnaires; patient related information was obtained from standardized BU01 forms instead. The current versions of BU01 forms however, do not contain information required for analysis of risk factors to contract the disease (e.g. information on living conditions and contact with water bodies); therefore only baseline data were available for analysis.

As PCR assessment was conducted in an external reference laboratory in Germany, a maximum number of samples were collected per patient to increase the probability for laboratory confirmation and to avoid repeated shipping of samples. To comply with recent WHO recommendations [30], future routine clinical management in Togo will have to reduce the number of diagnostic samples.

Concerning microscopy, beside a low concordance rate and a high percentage of false negative results, for approximately 30% of the patients with suspected BUD microscopy had either not been performed, local results could not be retrieved retrospectively, or a considerable number of slides had been discarded, thus were not available for re-checking at DITM. Improvement of the performance of microscopy requires a more stringent system for external quality assurance including regular supervision of local microscopy laboratories.

Although in general - with a turnaround time of approximately two weeks between shipment of samples and availability of laboratory results - PCR assessment at an external reference laboratory in Germany worked satisfactorily, local PCR capacities are desirable. Therefore, in January 2011 the National Hygiene Institute (INH) in Lomé has assumed the role of a National Reference Laboratory for PCR confirmation and microscopy.
